# The Clinical Importance of Measurement of Hematological Indices in the Breast Cancer Survivals: A Comparison Between Premenopausal and Postmenopausal Women

**DOI:** 10.14740/wjon956e

**Published:** 2016-04-03

**Authors:** Zhian M. I. Dezayee, Marwan S. M. Al-Nimer

**Affiliations:** a Hawler University, College of Health Sciences, Erbil, Iraq; b Department of Pharmacology, College of Medicine, Al-Mustansiriya University, P.O. Box 14132, Baghdad, Iraq

**Keywords:** Breast cancer, Mean platelet volume, Red cell distribution width

## Abstract

**Background:**

Determination of the hematological indices is a useful prognostic laboratory investigation in the cancer research. The neutrophil to lymphocyte ratio (LNR), red cell distribution width (RDW) and the platelet distribution width (PDW) are useful markers for the prediction and the prognosis of breast cancer. The aims of this study were to assess the hematological indices in breast cancer women survivals and to show if there were significant differences in these indices between pre- and postmenopausal women.

**Methods:**

This observational study was carried out in the Nanakali Hospital in Erbil, Kurdistan region, Iraq. A total number of 120 women with breast cancer under different modalities of management were enrolled in this study. The patients were grouped into premenopausal (group I, n = 30) and postmenopausal (group II, n = 90) women and the hematological indices of all patients were determined.

**Results:**

Significant low hemoglobin levels and red cell counts were observed among group II compared with group I patients. Group II women had significant high values of RDW and mean platelet volume (MPV) (16.68 ± 2.51 and 9.980 ± 1.271) compared with group I (15.12 ± 2.27 and 9.535 ± 1.082). There were insignificant differences between group I and group II regarding the values of the PWD, plateletcrit (PCT), NLR and platelet to lymphocyte ratio (PLR).

**Conclusions:**

We conclude that the low hemoglobin levels, and the high RDW and PDW are significantly existing in postmenopausal compared with premenopausal survival women, indicating that there are specific hematological indices associated with the postmenopausal survival of the breast cancer.

## Introduction

Determinations of hematological indices are used in the assessment of the clinical entities related to the oncology [1]. There are four determinants of the blood platelet used in the clinical practice and they are helpful in the assessment of the pathological conditions [2]. They are platelets count, mean platelet volume (MPV), platelet distribution width (PDW) and plateletcrit (PCT). The neutrophil to lymphocyte ratio (NLR) is a useful marker for the prediction and the prognosis of breast cancer [3]. Red cell distribution width (RDW) is also used in parallel with the PDW in the assessment of breast cancer [4].

In invasive breast cancer, the pretreatment value of the MPV is significantly higher than post-treatment value and its value is significantly correlated with the primary tumor size and local or distant metastasis, suggesting that MPV is a good prognostic marker [5]. In postmenopausal breast cancer women treated with tamoxifen, an endocrine therapy, the value of MPV was significantly higher than the corresponding value at pretreatment level (8.97 fL versus 8.2 fL) [6].

A lower survival rate of breast cancer is significantly associated with high NLR (> 2.57) and RDW (> 13.45%) and the latter is significantly associated with a high RDW measurement [7]. Krenn-Pilko et al [8] found that the value of NLR ≥ 3.0 was associated with poor disease-free survivals of breast cancer women. In one study, it has been found that there is no relationship between NLR value and the clinicopathological factors of the breast cancer but a disease-free survival is shorter and a disease-relapse is higher among women with a high NLR [9]. Furthermore, a high NLR is a negative prognostic marker in breast cancer women and considered as a predictor for overall survival, disease-free survival and recurrence-free survival in those patients [10]. Breast cancer women who had a high NLR and platelet to lymphocyte ratio (PLR) at the pretreatment state carried a risk of a high mortality rate [11]. The rationale of this study is based on the fact that the hematological index values are influenced by hormonal disturbances whether of endogenous or exogenous origin in women with breast cancer. Therefore, the aims of this study were to assess the hematological indices in breast cancer women survivals and to show if there were significant differences in these indices between pre- and postmenopausal women.

## Patients and Methods

This observational study was carried out in the Nanakali Hospital in Erbil, Kurdistan region. The study was conducted according to the guidelines of the Declaration of Helsinki with approval from a local ethical review board. A consent form was obtained from each patient before enrollment into the study. The criteria of inclusion were the survivals of breast cancer under different modalities of management. The present study excluded the patients with a history of rheumatic conditions, hematological, neoplastic, renal, hepatic or thyroid diseases, or patients receiving treatment with anti-inflammatory drugs.

The patients were grouped according to the status of menopause: group I (n = 30): premenopausal women; group II (n = 90): postmenopausal women.

Demographic data, medical history and treatment were collected in the center. Modifiable risk factors, events or complications, and current therapy were recorded. A person who reported smoking on admission was defined as current smoker. A venous blood sample was obtained from each patient and collected in the EDTA (anticoagulant) tube for measurement of hematological indices using the Coulter machine (HmX Hematology Analyzer with Autoloader, Beckman Coulter, Inc., USA). The hematological indices included hemoglobin, red cell count, white cell count, differential white cells, platelet count, MPV, PCT, RWD and PDW. The NLR and PLR were calculated simply by dividing the number of neutrophil or platelet to the number of lymphocyte cells. The erythrocyte sedimentation rate was traditionally measured using the Westergren method.

### Statistical analysis

Data were expressed as number, percent, and mean ± SD. Unpaired Student’s *t*-test and differences between percentages were used to evaluate differences between the two. For all tests, a two-tailed P ≤ 0.05 was considered statistically significant. All calculations were made using Excel 2003 program for Windows.

## Results

Table 1 shows the characteristics of the patients. Postmenopausal women were significantly older than corresponding premenopausal women. There was an insignificant difference in residency, marital status, history of current smoking and oral contraceptive pills intake, and a family history of breast cancer. Screening of the hematological indices revealed significant low red blood cell count and hemoglobin levels, while RWD and PWD values were significantly higher in postmenopausal women compared with premenopausal women (Table 2). The MPV, PCT, NLR and PLR values were non-significantly higher among postmenopausal women compared with premenopausal women (Table 2).

**Table 1:** Characteristics of the Patients Enrolled in the Study

	Premenopausal (n = 30)	Postmenopausal (n = 90)	Probability
Age (years)	42.8 ± 4.1	52.3 ± 4.1	0.000*
Marital status			
Single	6 (20)	15 (16.7)	
Married	24 (80)	75 (83.3)	0.677
Residency			
Urban	23 (76.7)	76 (84.4)	
Rural	7 (23.3)	14 (15.6)	0.331
Current smoking	10 (33.3)	26 (28.9)	0.645
Family history	14 (46.7)	44 (48.9)	0.832
Oral contraceptive	0 (0)	3 (3.33)	0.311

The results are expressed as number (%) and mean ± SD. *Significant difference between pre- and postmenopausal women.

**Table 2:** Data Analysis of Hematological Indices

Hematological indices	Premenopausal (n = 30)	Postmenopausal (n = 90)	Probability
Erythrocyte sedimentation rate (mm/h)	30.6 ± 14.3	30.2 ± 12.2	0.910
White blood cells (× 10^9^/L)	11.9 ± 2.5	12.6 ± 2.2	0.206
Lymphocytes (%)	51.5 ± 5.5	50.9 ± 5.9	0.625
Monocytes (%)	0.37 ± 0.30	0.34 ± 0.3	0.579
Neutrophils (%)	46.6 ± 12.7	48.0 ± 13.1	0.597
Red blood cells (× 10^12^/L)	4.901 ± 0.467	4.073 ± 0.554	0.0000*
Hemoglobin (g/dL)	11.4 ± 1.1	10.0 ± 1.5	0.0000*
Red cell distribution width (%)	15.12 ± 2.27	16.68 ± 2.51	0.00256*
Platelets count (× 10^9^/L)	286.0 ± 68.5	305.1 ± 66.4	0.203
Mean platelet volume (fL)	9.535 ± 1.082	9.980 ± 1.271	0.067
Platelet distribution width (fL)	12.348 ± 2.216	13.4 ± 2.923	0.04306*
Plateletcrit (%)	0.22 ± 0.11	0.26 ± 0.12	0.111
Neutrophil/lymphocytes ratio	0.912 ± 0.270	0.964 ± 10.33	0.397
Platelets/lymphocytes ratio	47.98 ± 11.65	48.39 ± 9.14	0.863

The results are expressed as mean ± SD. *Significant difference between pre- and postmenopausal women.

## Discussion

The results of this study show that postmenopausal women with breast cancer have a significant RDW and PDW compared with premenopausal women. There is no significant difference between pre- and postmenopausal women about the characteristics data at the time of the entry into the study. Therefore, these factors do not bias the results observed in this study. It is expected that the age of postmenopausal women is significantly higher than the corresponding age of the premenopausal women. Low hemoglobin level is reported in elderly patients with breast cancer treated with different modalities of therapeutic tools and this explains the significant low hemoglobin levels among postmenopausal patients [12]. Chemotherapeutic agents prescribed to the patients are also responsible for the low levels of red cell counts and hemoglobin as mentioned in recent studies [13]. Moreover, the low hemoglobin levels in cancer patients are of multifactorial etiology, in which nutritional, inflammatory, metabolic, immunological, radiotherapy and cytotoxic medications are involved in its pathogenesis [14]. Postmenopausal women have a significant high RDW compared with premenopausal women, indicating that the patients may carry a high rate of morbidity as Riedl et al [15] reported that RDW is an independent risk factor of poor survival in cancer. The results of the study by Seretis et al [16] showed that RDW is a marker of breast cancer activity as a significant high level found in breast cancer compared with breast fibroadenoma. Therefore, the results of this study show that the postmenopausal women may have active breast cancer compared with premenopausal women. A recent study demonstrates that a high PDW value is an independent predictive marker of breast cancer [1]. The results of this study are the high PDW value observed in breast cancer and the significant high levels observed in postmenopausal women. Moreover, Okuturlar et al [1] demonstrated that a cut-off value of NLR of 2.56 is considered as a prediction of breast cancer while this study shows that the mean value of NLR does not reach the cut-off value. Therefore, the importance of measuring the NLR in established and treated breast cancer is less useful. Furthermore, recent studies demonstrate that NLR is a significant independent marker of a poor prognosis for the subtype triple negative breast cancer [17]. An insignificant difference between premenopausal and postmenopausal women in a PLR indicates that the PLR is not a useful marker. Ulas et al [9] found that PLR is not a useful marker to assess the breast cancer free survival or overall survival. Limitations of the study are the variations in the breast cancer subtypes, the type of the cytotoxic drug regimen, and the other risk factors of breast cancer. However, in conclusion, the low hemoglobin levels, and the high RDW and PDW are significantly existing in postmenopausal compared with premenopausal survival women, indicating that there are specific hematological indices associated with the postmenopausal survival of the breast cancer patients.
